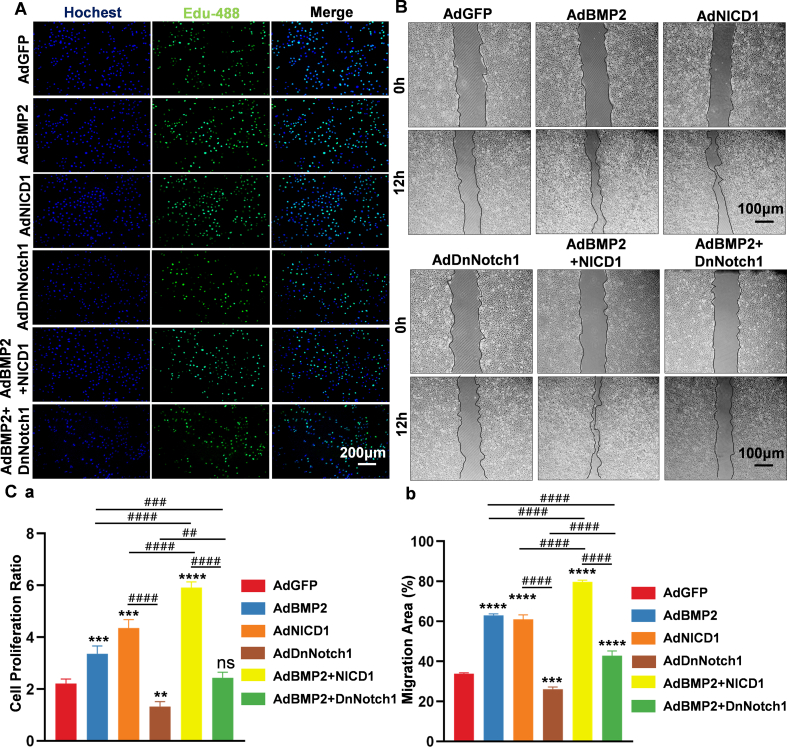# Notch1 signaling regulates Sox9 and VEGFA expression and governs BMP2-induced endochondral ossification of mesenchymal stem cells

**DOI:** 10.1016/j.gendis.2024.101336

**Published:** 2024-05-20

**Authors:** Jing Zou, Chengcheng Du, Senrui Liu, Piao Zhao, Shengqiang Gao, Bowen Chen, Xiangdong Wu, Wei Huang, Zhenglin Zhu, Junyi Liao

**Affiliations:** a Department of Orthopaedic Surgery, The First Affiliated Hospital of Chongqing Medical University, Chongqing 400016, China; b Chongqing Health Commission Key Laboratory of Motor System Regenerative and Translational Medicine, Orthopaedic Research Laboratory of Chongqing Medical University, Chongqing 400016, China; c Molecular Oncology Laboratory, Department of Orthopaedic Surgery and Rehabilitation Medicine, The University of Chicago Medical Center, Chicago, IL 60637, USA; d Department of Orthopaedic Surgery, Peking Union Medical College Hospital, Chinese Academy of Medical Sciences & Peking Union Medical College, Beijing 100730, China

**Keywords:** BMP2, Chondrogenic differentiation, Endochondral ossification, Mesenchymal stem cells, Notch1 signaling

## Abstract

Although bone morphogenetic protein 2 (BMP2) can induce chondrogenic differentiation of mesenchymal stem cells (MSCs), its induction of endochondral ossification limits the application of BMP2-based cartilage regeneration. Here, we clarified the mechanisms of BMP2-induced endochondral ossification of MSCs. *In vitro* and *in vivo* chondrogenic, osteogenic, and angiogenic differentiation models of MSCs were constructed. The expression of target genes was identified at both protein and mRNA levels. RNA sequencing, molecular docking, co-immunoprecipitation, and chromatin immunoprecipitation followed by sequencing were applied to investigate the molecular mechanisms. We found that BMP2 up-regulated the expression of Notch receptors and ligands in MSCs. Notch1 signaling activation was related to inhibition of chondrogenic differentiation, promotion of osteogenic and angiogenic differentiation. *In vivo* ectopic stem cell implantation identified that Notch1 signaling activation blocked BMP2-induced chondrogenesis and facilitated endochondral ossification of MSCs. Mechanistically, we elucidated Notch1 intracellular domain (NICD1)-RBPjk complex binding to SRY-box transcription factor 9 (Sox9) and vascular endothelial growth factor A (VEGFA) promoters to decrease Sox9 expression and increase VEGFA expression. These findings suggest that Notch1 signaling can regulate BMP2-induced endochondral ossification by promoting RBPjk-mediated Sox9 inactivation and VEGFA expression. It is conceivable that targeting Notch1 signaling mediated endochondral ossification would benefit BMP2-based cartilage regeneration.

## Introduction

Cartilage injuries are a prevalent clinical issue that can significantly impact quality of life. Because of their limited capacity for self-repair, such injuries often progress to osteoarthritis, which requires knee arthroplasty and thereby imposes a substantial financial burden on healthcare systems.^1^ Hence, cartilage regeneration holds a significant promise for addressing cartilage injuries.^1^, ^2^, ^3^ Bone morphogenetic protein 2 (BMP2) belongs to the transforming growth factor-beta (TGF-β) supper family and can induce mesenchymal stem cell (MSC) differentiation into various cell types of osteocytes, including chondrocytes, adipocytes, and endothelial cells.^4^, ^5^, ^6^ Mechanistically, BMP2 has been shown to effectively induce chondrogenic differentiation and inhibit osteogenic differentiation of MSCs by up-regulating the transcription factor Sox9 (SRY-box transcription factor 9).^7^^,^^8^ However, BMP2 also initiates hypertrophic differentiation and endochondral ossification by regulating downstream targets and engaging in crosstalk with other signaling pathways.^9^ Thus, elucidating the mechanisms underlying BMP2-induced endochondral ossification of MSCs is meaningful for BMP2-mediated cartilage regeneration.

Briefly, endochondral ossification involves chondrogenically differentiated MSCs forming an initial cartilaginous template, followed by hypertrophic and angiogenic differentiation. This process leads to vascularization and remodeling of the cartilaginous template, ultimately resulting in the formation of newly deposited bone tissue.^10^^,^^11^ We have previously elucidated that RUNX family transcription factor 2 (Runx2) serves as the key transcription factor that mediates BMP2-induced osteogenic and hypertrophic differentiation,^12^^,^^13^ however, the specific mechanisms of BMP2-induced angiogenic differentiation of MSCs are still unresolved. Previous studies have suggested the potential involvement of Notch signaling, a highly conserved pathway known to regulate angiogenic differentiation of MSCs and sprouting of endothelial tip cells and angiogenesis.^14^, ^15^, ^16^, ^17^ Mammals contain five Notch ligands (Delta (Dll) 1,3,4, and Jagged1/2) and four Notch receptors (Notch1–4).^18^, ^19^, ^20^, ^21^ Dll4-Notch1 was first identified as a regulator of sprouting angiogenesis. Knocking out Dll4 was found to be lethal from disruption of angiogenesis.^22^ Simultaneously, Jagged1 also participates in this process by binding to Notch1.^14^ These findings highlight the crucial role of Notch1 in hematopoiesis, emphasizing its essential function in blood vessels. Furthermore, Notch1 signaling is indispensable for the proper proliferation of chondrocyte progenitors and normal progression of hypertrophic chondrocyte differentiation into bone.^14^ Haller R et al discovered that Notch1 plays a significant role during the early stages of chondrogenic lineage determination by regulating Sox9 expression.^7^ Taken together, these studies indicate that Notch1 signaling is a pivotal component of MSC chondrogenic differentiation and endochondral ossification, highlighting the significance of Notch1 in BMP2-mediated control of these processes.

In this study, we used MSCs both *in vivo* and *in vitro* differentiation models to investigate the role and significance of Notch1 in regulating BMP2-induced chondrogenic differentiation and endochondral ossification. Our findings revealed that Notch1 was crucially involved in mediating the BMP2-induced endochondral ossification of MSCs. Mechanistically, we observed that activated Notch1 signaling facilitated the formation of the NICD1 (Notch1 intracellular domain)-RBPjk (recombination signal-binding protein for immunoglobulin kappa J region) complex, which subsequently bound to the Sox9 and vascular endothelial growth factor A (VEGFA) gene promoters. This interaction inhibited Sox9-mediated maintenance of chondrocyte phenotype while promoting VEGFA-mediated angiogenesis. These insights may offer novel perspectives for BMP2-mediated cartilage regeneration.

## Materials and methods

### Animal ethics statement

All animal surgeries were conducted under appropriate anesthesia. Animals were housed in standard cages, and surgical procedures were strictly adhered to animal welfare standards. Postoperatively, animals were provided with anesthesia for recovery to ensure their well-being. Euthanasia of the mice was performed at designated time points via intraperitoneal overdosage injection of pentobarbital sodium. Every effort was made to minimize animal suffering. Euthanasia was confirmed once the mice displayed no respiration, no heartbeat, or dilated pupils. Subsequently, ectopic masses were harvested from the injection site on the nude mice.

### Cell culture and chemicals

Human embryonic kidney (HEK)-293 cells, the mouse embryonic-derived MSC C3H10T1/2 cell line, and human umbilical vein endothelial cells (HUVECs) were procured from Pricella. The cells were cultured in a complete medium composed of 10% fetal bovine serum (Gibco, Australia) in Dulbecco's modified Eagle medium (Hyclone, China) supplemented with 100 U/mL penicillin and 100 mg/mL streptomycin. All cells were maintained in a standard cell culture incubator with 5% CO_2_ at 37 °C. Unless specified otherwise, all reagents were obtained from Sigma–Aldrich or Corning.

### Construction and production of recombinant adenovirus vectors

As outlined in our previous research, recombinant adenovirus vectors AdBMP2, AdNICD1, AdSox9, and AdDnNotch1 were generated using the AdEasy technology.^16^^,^^23^, ^24^, ^25^ Briefly, the coding regions of human BMP2, NICD1, Sox9, and the extracellular domain with the transmembrane region (aa #1–aa #1705) of mouse Notch1 were amplified and individually subcloned into a shuttle adenoviral vector, which generated recombinant adenoviral vectors. HEK-293 cell line was used for generating high titer recombinant adenovirus as described previously.^15^^,^^16^^,^^26^, ^27^, ^28^, ^29^ Additionally, green and red fluorescent protein (GFP and RFP) adenovirus vectors were used as mock virus controls.

### RNA isolation and quantitative reverse transcription PCR (qRT-PCR)

At the indicated time points, cell culture samples were processed into single-cell suspensions. Total RNA was extracted and purified using an RNA extraction kit (AG21013, Accurate Biology) following the manufacturer's instructions. Subsequently, total RNA was reverse transcribed using a reverse transcription reagent kit (RT Master Mix for qPCR, HY-K0510A, MCE). The cDNA was diluted and mixed with SYBR Green qPCR Master Mix (HY–K0501, MCE) and specific primers before qRT-PCR analysis. Quantitative PCR analysis was performed using the CFX96 real-time PCR detection system (Bio-Rad, USA). The real-time qRT-PCR program was as follows: 95 °C for 30 s, 95 °C for 5 s, 60 °C for 30 s, and repeated for 40 cycles. GAPDH was used as a reference gene. The 2^−ΔΔCt^ method was employed to normalize all sample expression values to GAPDH expression. PCR primer sequences are listed in Table S1.

### Protein harvesting and Western blot analysis

Total cellular proteins were extracted using RIPA lysis buffer (P0013k, Beyotime, China) containing 100 mM Tris–HCl, 100 mM β-mercaptoethanol, and protease and phosphatase inhibitors following the manufacturer's instructions. The protein concentration was measured using a BCA protein analysis kit (P0010S, Beyotime, China). Subsequently, the protein samples were denatured by boiling in SDS-PAGE protein loading buffer (P0015L, Beyotime, China) for 10 min and stored at −80 °C. Proteins were separated by electrophoresis using a precast protein gel (4%–20%, ACE, China) according to the standard protocol and transferred onto a polyvinylidene fluoride (PVDF) membrane (Millipore, USA) using a rapid transfer solution (WB4600, NCM Biotech, China). The PVDF membrane was then blocked with a rapid blocking buffer (P30500, NCM Biotech, China) for 1 h and incubated with specific primary antibodies against Sox9 (380995, ZENBIO), Col2a1 (collagen type II alpha 1 chain; BAO533, BOSTER), Runx2 (#12556, CST), Col1a1 (collagen type I alpha 1 chain; R26615, ZENBIO), OPN (osteopontin; A5427, BIMAKE), Notch1 (ab52627, Abcam), BMP2 (YT5651, Immunoway), NICD1 (#4147 CST), RBPjk (14613-1-AP Protenich), and GAPDH (R24404, ZENBIO) overnight and washed three times (10 min per wash) in Tris-buffered saline with Tween-20 on a shaker. Finally, the membranes were incubated with appropriate horseradish peroxidase (HRP) secondary antibody (MBW112, Mengbio) and visualized using Immobilon western chemiluminescent HRP substrate (Millipore, USA). Relative protein expression was analyzed using Image J software with GAPDH as a control.

### Alkaline phosphatase (ALP) activity

C3H10T1/2 cells were seeded in 24-well plates at a 40% confluence and transfected with the corresponding adenoviruses according to the groups. At specified time points, ALP staining was performed using the BCIP/NBT Alkaline Phosphatase Color Development Kit (C3206, Beyotime) following the manufacturer's instructions. After 30 min, cells were examined under an inverted microscope, and their images were captured using a high-resolution camera.^30^ As for quantitative ALP activity, cells were lysed by lysis reagent (Promega, USA). Then, ALP activity was measured with a Thermo Scientific (USA™) kit following the manufacturer's protocol. In addition, the protein concentration of cell lysates was determined by the BCA protein detection kit (P0010S, Beyotime, China), and the ALP activity was normalized to total protein among samples.

### Alizarin red S staining

Following cell culture and adenovirus transfection as described above, when the cell density reached 80%–90%, the culture medium was changed to osteogenic differentiation medium (PD-003, Procell, China) and cells were cultured for 14 days. As mentioned previously, mineralized nodules were assessed through Alizarin red S staining as previously characterized.^31^ Briefly, cells were fixed with 1% glutaraldehyde for 10 min and then washed with phosphate buffer saline solution (PBS), followed by incubation with 2% alizarin red S staining at room temperature for 30 min. The staining of calcium mineral deposits was documented under bright field microscopy and a high-resolution camera after washing with acidic PBS (pH = 4.2). For quantification, alizarin red S was dissolved in 10% acetic acid and the absorbance was detected at 405 nm with an enzyme label. The total DNA was purified from each cell well using TRIzol and measured with a spectrophotometer (Thermo, NanoDrop). The results were normalized to total DNA per well.

### Alcian blue staining

At indicated time points, cells were washed with PBS, fixed with 4% paraformaldehyde for 30 min, and again washed with PBS. Samples were subjected to 0.5% alcian blue dye (G1027-100 ML, Servicebio) for 20 min and then acidic alcohol to remove unbound dye. Samples were observed under an inverted microscope and images were captured using a high-resolution camera.^9^

### Cell migration and wound healing assays

Cell migration was detected with Transwell plates. C3H10T1/2 cells infected with indicated adenovirus were seeded in the lower chambers of Transwell plates (24-well plates, 8.0 μm, Jet Bio-Filtration) at a 40% confluence. When the cell density reached 80%, the medium was removed and the cells were washed with PBS three times. Then, a fresh complete medium containing 5% fetal bovine serum was added. Simultaneously, 2.5 × 10^4^ HUVECs were seeded in the upper chambers of the Transwell. After co-culturing for 12 h, the upper chambers were taken out, and cells on the upper membrane were gently removed with a cotton swab. The upper chambers were fixed at room temperature for 20 min with 4% paraformaldehyde, Finally, cells on the lower membrane were stained using 0.1% crystal violet staining solution as previously characterized.^32^^,^^33^

As for the wound healing assay, HUVECs were seeded in the lower chambers of Transwell plates (6-well plates, 0.4 μm, Jet Bio-Filtration, China) and cultured to sub-confluence. Simultaneously, C3H10T1/2 cells transfected with different adenovirus were seeded in the upper chambers of Transwell plates at a 40% confluence. When the cell density reached 80%, the medium was removed, and cells were washed three times with PBS, followed by replacement with a fresh complete medium. Using a 200 μL pipette tip, HUVEC cells were scraped vertically along the diameter of the 6-well plate, and C3H10T1/2 cells prepared as mentioned above were placed in the upper chambers of Transwell plates for co-culture. After 12 h, the areas of the scratches in each group were recorded, and the change in scratch area was calculated using Image J software.^34^

### Cell proliferation assay

C3H10T1/2 cells transfected with different adenovirus vectors and HUVECs were co-cultured through Transwell plates (6-well plates, 0.4 μm, Jet Bio-Filtration, China). The initial cell density of seeded HUVECs was approximately 40%. After 24 h of co-culture, cell proliferation of HUVECs was assessed using the EdU-488 cell proliferation assay kit (C0071S, Beyotime). Proliferating cells were detected under an inverted microscope and quantified using Image J software.^35^

### Tube formation assay

C3H10T1/2 cells and HUVECs were co-cultured in Transwell plates (24-well plates, 0.4 μm, Jet Bio-Filtration, China) for tube formation experiments. C3H10T1/2 cells were seeded in the upper chamber of the 24-well Transwell plate. After cell density and viral infection were appropriate, matrix gel and sterile tips were pre-chilled at 4 °C overnight. Subsequently, 100 μL of matrix gel (356230, CORNING) mixed with 100 μL of PBS suspension was painted on the lower chamber of the Transwell plate. HUVECs were then replated in the lower chamber at a density of 2 × 10^5^ cells. After co-culturing for 3 h, images were captured using an inverted microscope, and the quantification of tube formation was analyzed using Image J.^36^, ^37^, ^38^

### Enzyme-linked immunosorbent assays (ELISA)

According to the manufacturer's instructions, ELISA kits (for vascular endothelial growth factor/VEGF, epidermal growth factor/EGF, Von Willebrand factor/vWF, Jubang Biological, China) were used to bind the test samples with specific antibodies inside microplate wells. Then, enzyme-labeled secondary antibodies were added, followed by another washing step. A substrate was then added to generate a measurable signal, and the concentration of the target protein was determined by measuring the optical density (OD) values at 450 nm.

### Subcutaneous stem cell implantation

The Institutional Animal Care and Use Committee approved the use and care of animals in this study. All experimental procedures were conducted following approved guidelines. C3H10T1/2 cells were infected with adenovirus according to their respective groups. Twenty-four hours after infection, the cells were harvested and resuspended in PBS containing 300 U/mL penicillin and 300 mg/mL streptomycin at a concentration of 5 × 10^6^ cells per 100 μL. Subsequently, the cell suspension was injected subcutaneously into the lateral abdomen of athymic nude mice (*n* = 3/group, female, 4–5 weeks old). At the indicated time points, the animals were euthanized, and tissue blocks were retrieved from the injection site. Ectopic nodules were fixed in 4% paraformaldehyde (P0099-100 ml, Beyotime) at room temperature for 24 h and then decalcified in ethylene diamine tetraacetic acid (EDTA) decalcification solution (G1105-500 ML, Servicebio). The decalcification solution was changed every 2–3 days until the tissue samples were completely softened. Samples were then embedded in paraffin. Continuous 5 μm-thick sections were obtained and subjected to histological staining.

### Hematoxylin-eosin staining and saffranine O-solid green staining

The paraffin-embedded sections were deparaffinized in xylene and then rehydrated through graded ethanol. Subsequently, dewaxed samples were stained with hematoxylin-eosin (G1120, solarbio) and safranine O-solid green (G1371, solarbio) according to the manufacturer's instructions as previously described.^16^ The sections were photographed using an upright microscope, and the images were histologically evaluated. The relative trabecular bone area was analyzed using Image J software. Blood vessels were counted in high-power fields under double-blind conditions by three experts.^24^

### Immunohistochemistry staining

Immunohistochemical staining was done as previously described.^15^^,^^39^ Briefly, tissue sections were incubated with the corresponding primary antibodies (COL1A1, 66761-1-Ig and CD31, 28083-1-AP, Proteintech) at 4 °C overnight. After washing with PBS three times, the sections were incubated with biotinylated secondary antibodies (SP0041, Solarbio) for 30 min, followed by incubation with avidin–biotin–peroxidase complex (HRP) at room temperature for 20 min. The immunohistochemistry results of immunohistochemistry were quantified using Image J software.

### RNA sequencing and bioinformatics analysis

Total RNA extracted from C3H10T1/2 cells infected with different adenovirus was subjected to RNA sequencing (Unichuan Biotechnology, Hangzhou, China). Samples were double-ended sequenced using Illumina NovaseqTM 6000 (LC Bio Technology CO., Ltd. Hangzhou, China) according to standard procedures in PE150 sequencing mode. The R Programming Language was used to analyze the raw data and identify differentially expressed genes. The differential expression transcripts with a *p*-value ≤0.05 and fold change ≥2 were selected for enrichment analysis of biological function (Gene Ontology, GO) and signaling pathway of gene ontology (Kyoto Encyclopedia of Genes and Genomes, KEGG).

### Target prediction and molecular docking

The RBPJK (UniProt ID: P31266), Notch1 (UniProt ID: Q01705), and MAML-1 (mastermind-like transcriptional coactivator 1; UniProt ID: Q6T264) protein sequence information was obtained from the UniProt database. AlphaFold2 was utilized to predict the three-dimensional structure of these proteins. The transmembrane region of Notch1 was identified using DeepTMHMM, with the 1660–2517 amino acid region selected as the NICD structural domain.^40^ The Sox9 transcriptional promoter sequence was retrieved,^41^ and a model of the selected sequence was constructed using Discovery Studio. The complex model of RBPjk-NICD-MAML-1 was built by Discovery Studio, which was subsequently used for molecular docking with the Sox9 promoter employing Hdock. The complex with the highest score was selected for visual analysis.

### Co-immunoprecipitation

Following adenoviral transduction for 48 h in each group, C3H10T1/2 cells were collected and resuspended in an appropriate amount of cell lysis buffer (P0013, Beyotime) containing a protease inhibitor cocktail (P1005, Beyotime). The total protein was extracted by lysing the cells on ice for 30 min, followed by centrifugation at 12,000 rpm at 4 °C for 15 min. The supernatant was then collected. A small portion of the cell lysate was reserved for Western blot analysis as the input sample. For the remaining cell lysate, 1 μg of an anti-RBPJK antibody (14613-1-AP, Proteintech) was added and the mixture was gently shaken and incubated at 4 °C overnight. Subsequently, 10 μL of pre-treated protein A agarose beads (P2051-2 mL) was added to the cell lysate, and the mixture was incubated with gentle shaking at 4 °C for 2–4 h to allow the antibody to conjugate with the protein A agarose beads. After the immunoprecipitation reaction, the beads were washed 3–4 times with 1 mL of lysis buffer, and then 15 μL of 5 × SDS loading buffer was added to the beads. The mixture was boiled in a water bath for 10 min. Western blot analysis was performed to analyze the binding of the antibody to the RBPjk protein.

### Chromatin immunoprecipitation followed by sequencing (CHIP-seq)

As previously described,^15^ C3H10T1/2 cells infected with adenovirus were crosslinked with 1% formaldehyde at room temperature for 10 min, and 0.125 M glycine (HY–Y0966, MCE) was added to terminate the crosslinking reaction for 5 min. The cell suspension was sent to Chongqing Jingshi Biotechnology Co., Ltd. according to the instructions, the RBPjk protein and DNA fragments were pulled down by RBPjk antibody, and then RBPjk protein was confirmed by western blots. The DNA extracted from the precipitation was prepared in a high-throughput DNA sequencing library and was sequenced on a DNBSEQ-T7 sequencer (MGI Technology Co., Ltd.). The data was analyzed after the raw sequencing data was filtered using Trimmomatic (version 0.36).

### Statistical analysis

All quantitative experiments were performed in triplicate or repeated three times. Quantitative data were presented as mean ± standard deviation and analyzed using GraphPad Prism 9.0. Unpaired student's *t*-tests were used for two-group comparisons, while one-way or two-way analysis of variance (ANOVA) was used for multiple groups, followed by Tukey–Kramer tests. A *p*-value <0.05 was considered statistically significant. This statistical analysis allows for assessing the significance of observed differences and helps draw conclusions based on the data obtained from the experiments.

## Results

### The activation of Notch1 signaling during BMP2-induced differentiation of MSCs

C3H10T1/2 cells were infected with AdBMP2 and AdGFP was used as a control. As shown in Figure S1A, adenovirus efficiently infected the C3H10T1/2 cells. With the stimulation of BMP2, chondrogenic differentiation marker Col2a1 was up-regulated gradually from day 1 to day 7 and then back to baseline gradually from day 9 to day 11 (Fig. S1B *a*). Conversely, the osteogenic marker Col1a1 exhibited a slow increase in expression from day 1 to day 7 and a notable up-regulation from day 7 to day 11 (Fig. S1B *b*). These findings suggest that BMP2 can induce chondrogenic differentiation of MSCs at an early stage, followed by osteogenic differentiation. Simultaneously, we detected the expression of Notch receptors and ligands with the stimulation of BMP2. We observed up-regulated Notch receptor expression levels at the early stage (Fig. S1C *a*), while Notch1 and Jagged1 were significantly increased at the mid-to-late stages (Fig. S1C *b*) of BMP2-induced MSC differentiation (Fig. S1C). In addition, BMP2 dramatically up-regulated the expression of NICD1 and RBPjk at protein level on day 3 and day 7 respectively (Fig. S1D *a*, *b*, E *a–d*), which indicated the activation of Notch1 signaling with the stimulation of BMP2.

Based on these results, we deduced that Notch signaling, especially Notch1 signaling may mainly participate in BMP2-induced endochondral ossification of MSCs.

### Adenovirus-mediated gene expression of BMP2 and NICD1

C3H10T1/2 cells were infected with indicated adenovirus, AdBMP2 was used to induce MSC differentiation, AdNICD1 was used to up-regulate Notch1 signaling, and AdDnNID1 was used to down-regulate Notch1 signaling (Fig. S2). As depicted in Figure S2A and B, the adenoviruses effectively infected MSCs, as indicated by the observed fluorescence. We found that AdBMP2 effectively up-regulated BMP2 mRNA expression levels, while AdNICD1 did not affect the expression of BMP2. Accordingly, AdNICD1 significantly up-regulated the expression of NICD1 and synergistically potentiated BMP2-induced expression of NICD1 (Fig. S2A *b*). At the same time, AdDnNotch1 did not influence the expression of BMP2 and dramatically inhibited BMP2-induced NICD expression (Fig. S2B *b*). These results suggested that AdNICD1 could effectively potentiate BMP2-induced activation of Notch1 signaling and AdDnNotch1 could effectively suppress BMP2-induced activation of Notch1 signaling.

### Down-regulation of Notch1 signaling promoted BMP2-induced chondrogenic differentiation and inhibited BMP2-induced osteogenic differentiation of MSCs

To clarify the influence of down-regulation of Notch1 signaling in BMP2-induced MSC differentiation, both chondrogenic and osteogenic markers were detected. The results demonstrated that AdDnNotch1 significantly promoted BMP2-induced expression of the key chondrogenic differentiation transcription factor Sox9 and the chondrogenic differentiation marker Col2a1 compared with the control group at the mRNA level (Fig. 1A *a*, *b*). On the contrary, AdDnNotch1 significantly inhibited BMP2-induced expression of the key osteogenic differentiation transcription factor Runx2 and osteogenic differentiation markers Col1a1 and OPN at the mRNA level (Fig. 1A *c–e*). Alcian blue staining indicated that AdDnNotch1 significantly promoted BMP2-induced glycosaminoglycan synthesis compared with control groups (Fig. 1B). As for the activity of the early osteogenic differentiation marker ALP (Fig. 1C *a*, *b*) and deposition of the late osteogenic marker calcium (Fig. 1D *a*, *b*), we found that AdDnNotch1 significantly inhibited BMP2-induced early and late osteogenic differentiation of MSCs. Quantitative analysis of ALP activities on day 3 and day 7, and alizarin staining on day 14 were shown in Figure 1E *a–c*. Western blot showed that AdDnNotch1 mediated down-regulation of Notch1 potentiated BMP2-induced Sox9 and Col2a1 expression, and inhibited BMP2-induced Runx2, Col1a1, and OPN expression at the protein level (Fig. 1F). Quantitative analysis showed the same trend (Fig. 1F *b*).

**Figure 1:**
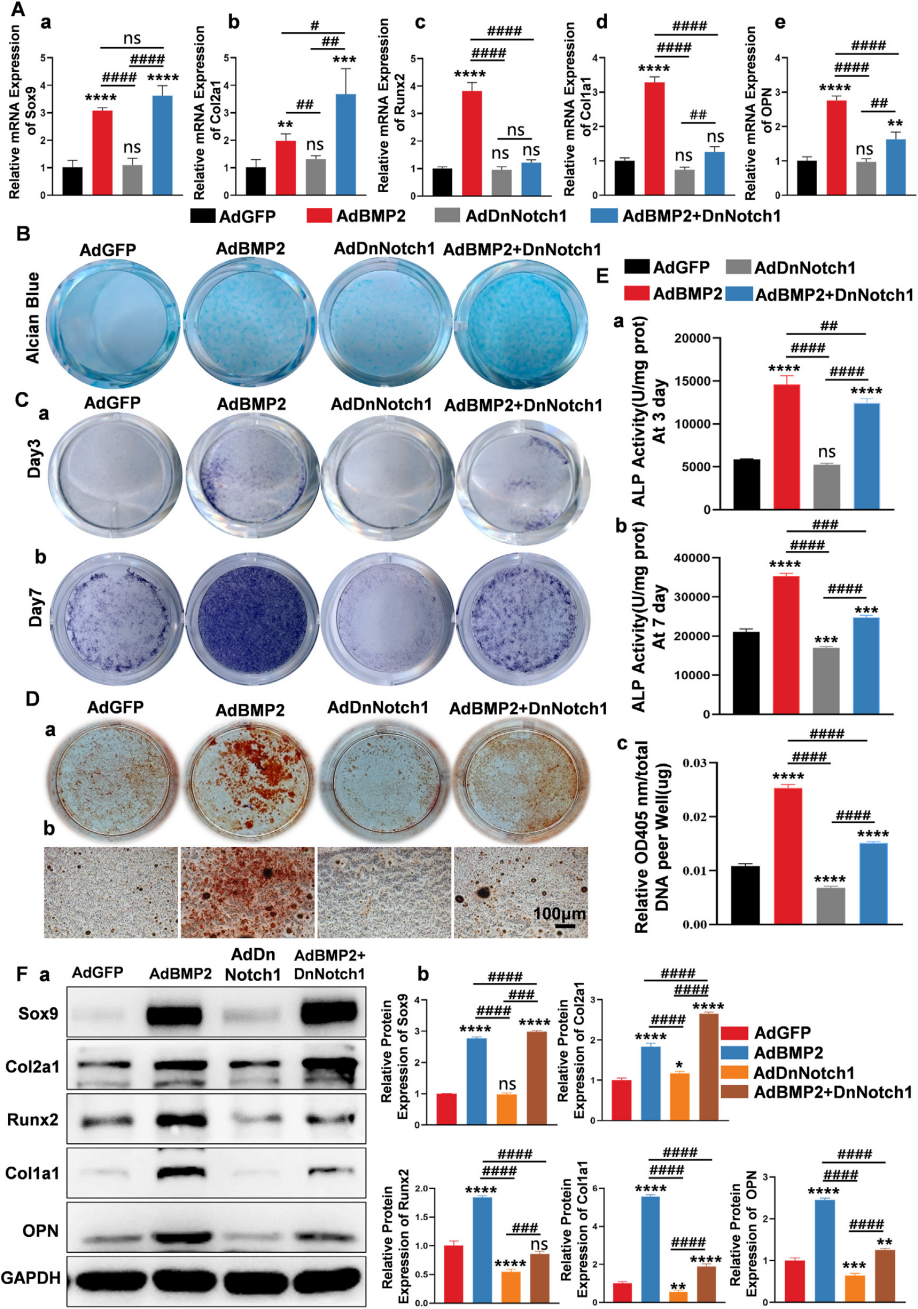
Down-regulation of Notch1 signaling promoted BMP2-induced chondrogenic differentiation and inhibited BMP2-induced osteogenic differentiation of MSCs *in vitro*. **(A)** C3H10T1/2 cells were transfected with AdGFP, AdBMP2, AdDnNotch1, and AdBMP2+DnNotch1, respectively. On day 7, quantitative reverse transcription PCR was used to detect the expression of chondrogenic differentiation markers (Sox9 and Col2a1) and osteoblastic differentiation markers (Runx2, Col1a1, and OPN) of MSCs. **(B)** To detect the expression of sulfated glycosaminoglycan during C3H10T1/2 cell differentiation, alcian blue staining was performed on day 7 after cells were transfected with recombinant adenovirus. **(C)** Alkaline phosphatase (ALP) staining experiments were used to determine ALP activity on day 3 **(*a*)** and day 7 **(*b*)** respectively. **(D)** For matrix mineralization, alizarin red S staining was performed on day 14 **(*a*)**; microscopic **(*b*)** observations showed that down-regulation of Notch1 signaling inhibited BMP2-induced calcium deposition. **(E)** Quantitative analysis of ALP activities and calcium deposition. The ALP activity was quantified at OD 405 nm and normalized by protein concentration per well (unit/mg protein) on day 3 **(*a*)** and day 7 **(*b*)**. Alizarin red staining was quantified at OD 405 nm and normalized to total DNA per well (OD_405_ nm/μg DNA) **(*c*)**. **(F)** Western blot analysis for the chondrogenic differentiation markers Sox9 and Col2a1 and the osteogenic markers Runx2, Col1a1, and OPN. Protein bands **(*a*)** and quantitative analysis **(*b*)**. The relative expression of Sox9, Col2a1, Runx2, Col1a1, and OPN proteins were analyzed using GAPDH as control **(*b*)**. One-way analysis of variance; ∗∗∗∗*p* < 0.0001, ∗∗∗*p* < 0.001, ∗∗*p* < 0.01, and ∗*p* < 0.05 versus the AdGFP group; ^####^*p* < 0.0001, ^###^*p* < 0.001, ^##^*p* < 0.01, and ^#^*p* < 0.05 versus the indicated group; ns, *p* > 0.05. BMP2, bone morphogenetic protein 2; Col1a1, collagen type I alpha 1 chain; Col2a1, collagen type II alpha 1 chain; MSC, mesenchymal stem cell; Notch1, Notch receptor 1; OPN, osteopontin; Runx2, RUNX family transcription factor 2; Sox9; SRY-box transcription factor 9.

### Up-regulation of Notch1 signaling attenuated BMP2-induced chondrogenic differentiation and enhanced BMP2-induced osteogenic differentiation of MSCs

We also explored the effect of up-regulating Noth1 signaling on BMP2-induced chondrogenic and osteogenic differentiation of MSCs. AdNICD1 was used to activate Notch1 signaling as previously characterized.^16^ We found that Notch1 signaling activation significantly inhibited BMP2-induced up-regulation of Sox9 and Col2a1 expression levels, while this activation dramatically enhanced BMP2-induced expression of Runx2, Col1a1, and OPN at the mRNA level (Fig. S3A). Alcian blue staining also indicated that activated Notch1 signaling significantly inhibited BMP2-induced glycosaminoglycan synthesis compared with the control groups (Fig. S3B). Assessments of the activity of the early osteogenic differentiation marker ALP (Fig. S3C *a*, *b*) and deposition of the late osteogenic marker calcium (Fig. S3D *a*, *b*) indicated that AdNICD1 significantly promoted early and late osteogenic differentiation of MSCs induced by BMP2. Quantitative analysis of ALP activities on day 3 and day 7, and alizarin staining on day 14 were shown in Figure S3E *a–c*. Western blot analysis showed that AdNCD1-mediated up-regulation of Notch1 signaling attenuated BMP2-induced Sox9 and Col2a1 protein expression levels, while dramatically enhancing BMP2-induced Ruxn2, Col1a1, and OPN expression at the protein level (Fig. S3E *a*). Quantitative analysis showed the same trend (Fig. S3E *b*).

### Notch1 signaling promoted BMP2-induced endochondral ossification *in vivo*

To further clarify the effects of Notch1 signaling on BMP2-induced MSC differentiation, subcutaneous MSC implantation was carried out. MSCs were infected with indicated adenovirus, and then ectopic masses were harvested at 4 weeks and 6 weeks (Fig. 2A). We found that BMP2 could induce MSC chondrogenic differentiation and subsequently trigger endochondral ossification (Fig. 2B, C). As shown in Figure 2C, BMP2-induced MSC chondrogenic differentiation with few blood vessels and trabecular bone formation at 4 weeks, and the chondrocytes were replaced by trabecular bone at 6 weeks (Fig. 2B, left). Conversely, with the down-regulation of Notch1 signaling, fewer blood vessels were formed at 4 weeks and less trabecular bone was formed at 6 weeks (Fig. 2B, middle). In contrast, when Notch1 signaling was activated, more blood vessels were formed at 4 weeks and more trabecular bone was formed at 6 weeks (Fig. 2B, right). Quantitative blood vessel numbers and trabecular bone volume analysis also indicated that down-regulation of Notch1 signaling could inhibit BMP2-induced blood vessels and trabecular bone formation, and up-regulation of Notch1 signaling promoted BMP2-induced blood vessels and trabecular bone formation (Fig. 2B *b*, C *b*).

**Figure 2:**
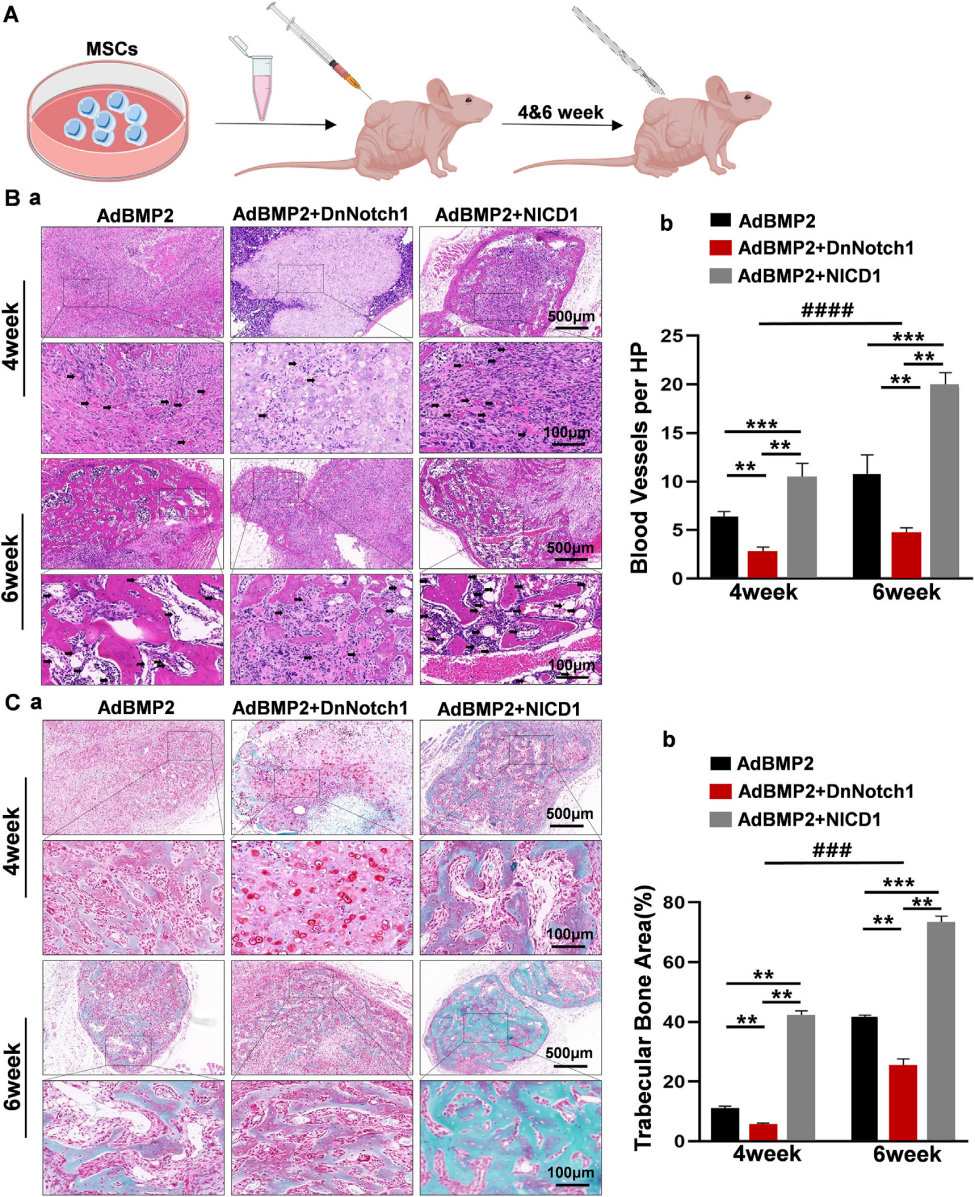
Notch1 signaling regulated BMP2-induced endochondral ossification of MSCs *in vivo*. **(A)** MSCs transfected with the corresponding adenovirus were transplanted subcutaneously into nude mice, and ectopic masses were removed at 4 weeks and 6 weeks, respectively. Created with Bio render.com. **(B)** Samples retrieved at 4 weeks and 6 weeks were stained with hematoxylin and eosin for histological analysis. The arrows indicate new blood vessels **(*a*)**. Quantitative analysis of high-power field blood vessels showed that down-regulation of Notch1 signaling inhibited BMP2-induced angiogenesis, while its up-regulation produced the opposite result **(*b*)**. **(C)** Saffranine O-solid green staining for detecting the formation of cartilage matrix and trabecular bone **(*a*)**. Quantitative analysis of trabecular bone area **(*b*)**. The scale bar is 500 μm at low power or 100 μm at high power. One-way analysis of variance; ∗∗∗*p* < 0.001 and ∗∗*p* < 0.01 versus the AdBMP2 group. Two-way analysis of variance; ^####^*p* < 0.0001 versus the 4-week group. BMP2, bone morphogenetic protein 2; MSC, mesenchymal stem cell; Notch1, Notch receptor 1.

Subsequently, we examined Col1a1 and CD31 protein expression with immunohistochemistry assays. We found that BMP2 could induce Col1a1 protein expression at 6 weeks. Down-regulation of Notch1 signaling inhibited BMP2-induced Col1a1 protein expression, while up-regulation of Notch1 signaling potentiated BMP2-induced Col1a1 protein expression at 4 weeks and 6 weeks (Fig. S4A *a*). Quantitative analysis supported these observed trends (Fig. S4A *b*). To investigate the effects on angiogenesis, we identified CD31 positive cells, finding that BMP2-stimulated CD31^+^ cell generation was attenuated by down-regulation of Notch1 signaling and potentiated by up-regulation of Notch1 signaling (Fig. S4B *a*). Quantitative analysis was consistent with these trends (Fig. S4B *b*).

These results indicated the regulatory role of Notch1 signaling in BMP2-induced endochondral ossification.

### Notch1 signaling regulated BMP2-induced angiogenic differentiation of MSCs by VEGFA activation

To address the effect of BMP2-induced angiogenic differentiation of MSCs on HUVEC migration, Transwells with 8 μm diameter mesh membranes were used for co-culturing HUVECs and MSCs infected with indicated adenovirus (Fig. 3A *a*). Following BMP2 stimulation, more HUVECs migrated from the upper side to the back of the upper chamber compared with the control group, indicating the angiogenic differentiation effects of BMP2 on MSCs. Upon up-regulation of Notch1 signaling with AdNICD1, a further increase in HUVEC migration was noted. However, down-regulation of Notch1 signaling with AdDnNotch1 led to a reduction in HUVEC migration compared with the AdBMP2 group (Fig. 3A *b*). Quantitative analysis corroborated these findings (Fig. 3A *c*). Additionally, HUVEC tube formation assays were conducted to confirm the angiogenic differentiation effects induced by BMP2. Transwells with 0.4 μm diameter mesh membranes were used to co-culture MSCs (upper chamber) infected with the indicated adenoviruses and HUVECs (lower chamber) (Fig. 3B *a*). The tube formation effects induced by BMP2 were enhanced by up-regulating Notch1 signaling and attenuated by down-regulating Notch1 signaling (Fig. 3B *b*). Quantitative analysis supported these observations (Fig. 3B *c*). Furthermore, ELISAs were used to examine the secretion of angiogenesis growth factor VEGFA, vWF, and EGF following BMP2 stimulation. The results revealed that BMP2-induced secretion of these factors was potentiated by up-regulation of Notch1 signaling and inhibited by down-regulation of Notch1 signaling (Fig. 3C *a–c*). In addition, VEGFA protein expression levels were assessed using Western blot analysis. Activating Notch1 signaling with AdNICD1 significantly potentiated BMP2-induced VEGFA protein expression (Fig. 3D *a*), while down-regulating Notch1 signaling with AdDnNotch1 dramatically inhibited BMP2-induced VEGFA protein expression (Fig. 3E *a*). Quantitative analysis of the Western blot bands confirmed these trends (Fig. 3D *b*, E *b*). A simultaneous co-culture system was employed to investigate the effects of BMP2-induced MSC angiogenic differentiation on HUVEC proliferation (Fig. S5A, C *a*) and migration (Fig. S5B, C *b*) rates, further supporting the role of Notch1 signaling in regulating BMP2-induced angiogenic differentiation of MSCs.

**Figure 3:**
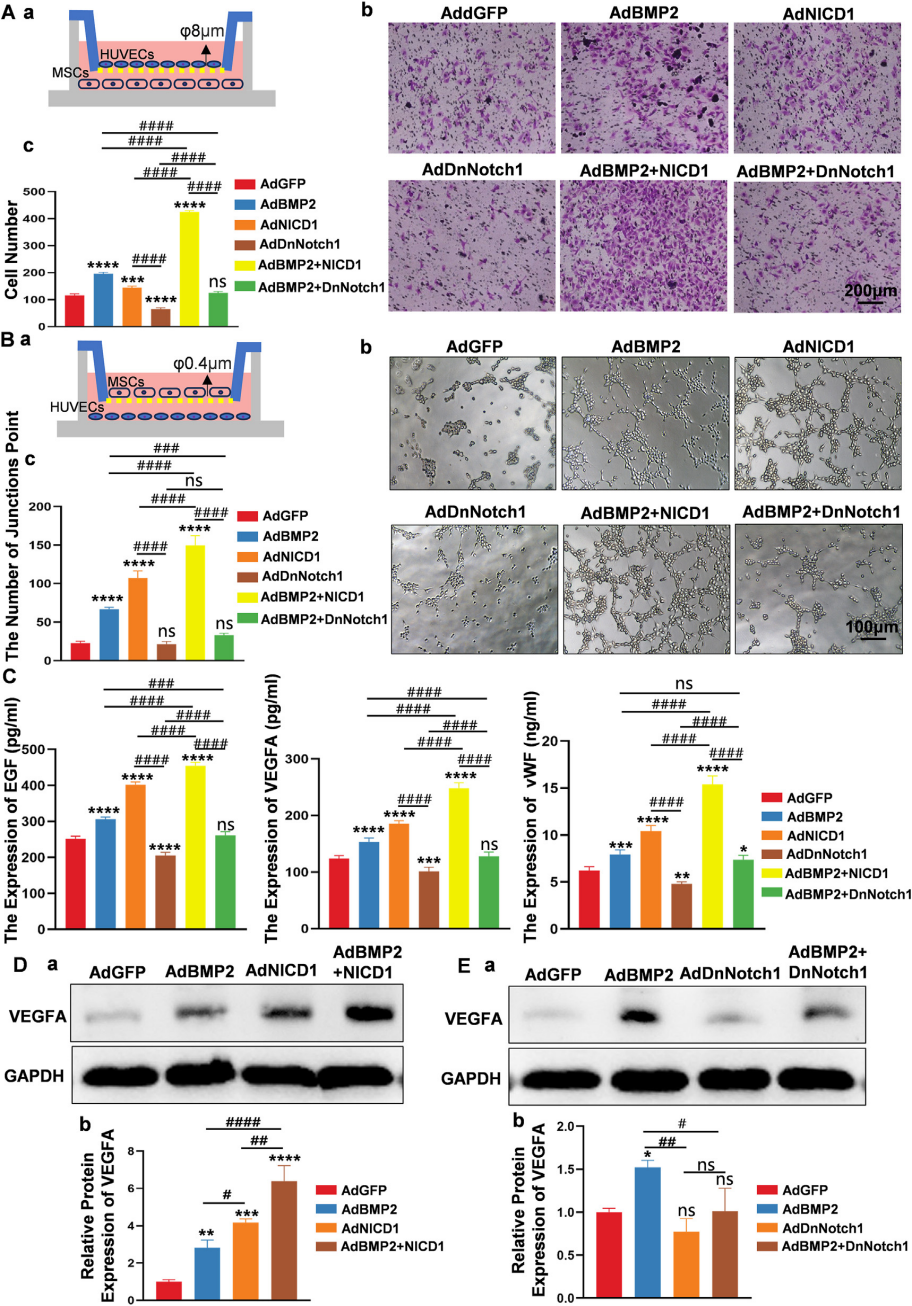
Notch1 signaling promoted BMP2-induced angiogenic differentiation of MSCs by activating VEGFA. **(A)** Notch 1 signaling regulated BMP2-induced HUVEC migration *in vitro*. MSC and HUVEC co-culture system was constructed **(*a*)**. MSCs infected with adenovirus were cultured in the lower compartment of Transwell, and HUVECs were cultured in the upper compartment with an 8 μm diameter mesh membrane. The HUVEC cells that penetrated the mesh membrane from the upper compartment were detected by crystal violet staining assay **(*b*)**. Scale bar = 200 μm. Quantitative analysis of the number of stained cells showed that BMP2-induced migration of HUVECs was enhanced by overexpression of NICD1 and inhibited by down-regulation of Notch1 signaling **(*c*)**. **(B)** Notch1 signaling regulated BMP2-induced HUVEC tubule formation *in vitro*. MSCs infected with adenovirus were cultured in the upper chamber of the co-culture plate with 0.4 μm diameter mesh membrane and HUVECs in the lower chamber **(*a*)**. The tubule-forming ability of HUVECs was detected by a tubule-forming experiment and recorded by microscope observation **(*b*)**. Scale bar = 100 μm. The number of tubule formation was quantitatively analyzed, and the results showed that overexpression of NICD1 enhanced the tubule formation capacity of BMP2-induced HUVECs, while down-regulation of Notch1 signaling decreased BMP2-induced tubule formation **(*c*)**. **(C)** The VEGFA **(*a*)**, vWF **(*b*)**, and EGF **(*c*)** secretion in the supernatant of MSCs transfected with indicated adenovirus. Quantitative analysis showed that NICD1 could enhance the secretion of the BMP2-induced angiogenic growth factors. **(D)** Exogenous activation of Notch1 signaling potentiated BMP2-induced VEGFA expression at the protein level. Quantitative analysis showed NICD1 enhanced BMP2-induced VEGFA expression. **(E)** Down-regulation of Notch1 signaling inhibited BMP2-induced VEGFA expression at the protein level. Quantitative analysis showed that DnNotch1 inhibited BMP2-induced VEGFA expression. One-way analysis of variance; ∗∗∗∗*p* < 0.0001, ∗∗∗*p* < 0.001, ∗∗*p* < 0.01, and ∗*p* < 0.05, versus the AdGFP group; ^####^*p* < 0.0001, ^###^*p* < 0.001, and ^##^*p* < 0.01 versus the indicated group; ns, *p* > 0.05. BMP2, bone morphogenetic protein 2; EGF, epidermal growth factor; HUVEC, human umbilical vein endothelial cell; MSC, mesenchymal stem cell; NICD1, Notch1 intracellular domain; Notch1, Notch receptor 1; VEGF, vascular endothelial growth factor; VEGFA, vascular endothelial growth factor A; vWF, Von Willebrand factor.

### NICD1 regulates BMP2-induced endochondral ossification by simultaneously inhibiting Sox9 and promoting VEGFA expression

*In vivo* and *in vitro* results indicated that Notch1 signaling attenuated BMP2-induced chondrogenic differentiation and promoted BMP2-induced endochondral ossification, however, the molecular mechanisms were not fully clear. To clarify the regulatory mechanisms of Notch1 signaling on BMP2-induced endochondral ossification, RNA sequencing was applied to detect differentially expressed genes. As shown in Figure 4A and B, there were 293 genes up-regulated and 708 genes down-regulated when compared between the AdBMP2 and AdBMP2+AdNICD1 groups. Among the differentially expressed genes, Sox9 was associated with BMP2-induced chondrogenic differentiation of MSCs. KEGG signaling enrichment analysis also indicated the TGF-β signaling, Wnt signaling, VEGFA signaling, and PI3K-Akt signaling pathways, among others, may be involved in this process. From our previous work, we first analyzed the expression of the key transcription factor Sox9. Fragments per kilobase of exon model per million mapped fragments (PFKM) analysis showed that BMP2-induced expression of Sox9 was inhibited following the activation of Notch1 signaling (Fig. 4D). These results indicated an interaction of NICD1 with Sox9. As a signaling transcription protein, RPBjk is a key protein that mediates NICD1 entering the nucleolus and regulating target gene expression. Therefore, we examined if RBPJK protein could bind to the Sox9 gene promoter. The three-dimensional conjunction of the Sox9 promoter and RBPjk is shown in Figure 4E *a*, *b*. Binding was predicted among RBPJK, NICD1, and MAML-1, as previously characterized.^40^ The three-dimensional conjunction of Sox9 promoter and RBPjk is shown in Figure 4F. According to the prediction, we selected the highest score in the top ten combinations, with a score of 560.50 Å. Sox9 promoter and RBPjk-NICD1-MAML-1 binding site were shown in Figure 4G *a*; hydrogen bind and van der Waals' force (VDW) were both found in the binding (Fig. 4G *b*, *c*). Next, co-immunoprecipitation experiments were used to confirm the binding between RBPjk and NICD1. We found that using RBPjk-marked bead selection, NICD1 protein could be detected in both the cell protein lysate (marked as input) and RBPjk-precipitated sample (marked as IP) (Fig. 5A). These results indicated that RBPJK could bind to NICD1 in these cells. Next, ChIP-seq was used to detect the binding between the Sox9 promoter region and RBPJK protein. RBPJK-precipitated products were detected by Western blot analysis, with IgG used as a negative control and the cell protein lysate marked as input (Fig. 5B). ChIP-seq data suggested enrichment of both the Sox9 and VEGFA promoter sequences in RBPjk-precipitated products (Fig. 5C). The Sox9 and VEGFA promoter sequences were each identified by quantitative PCR analysis, with IgG used as the control and the input% shown in Figure 5D.

**Figure 4:**
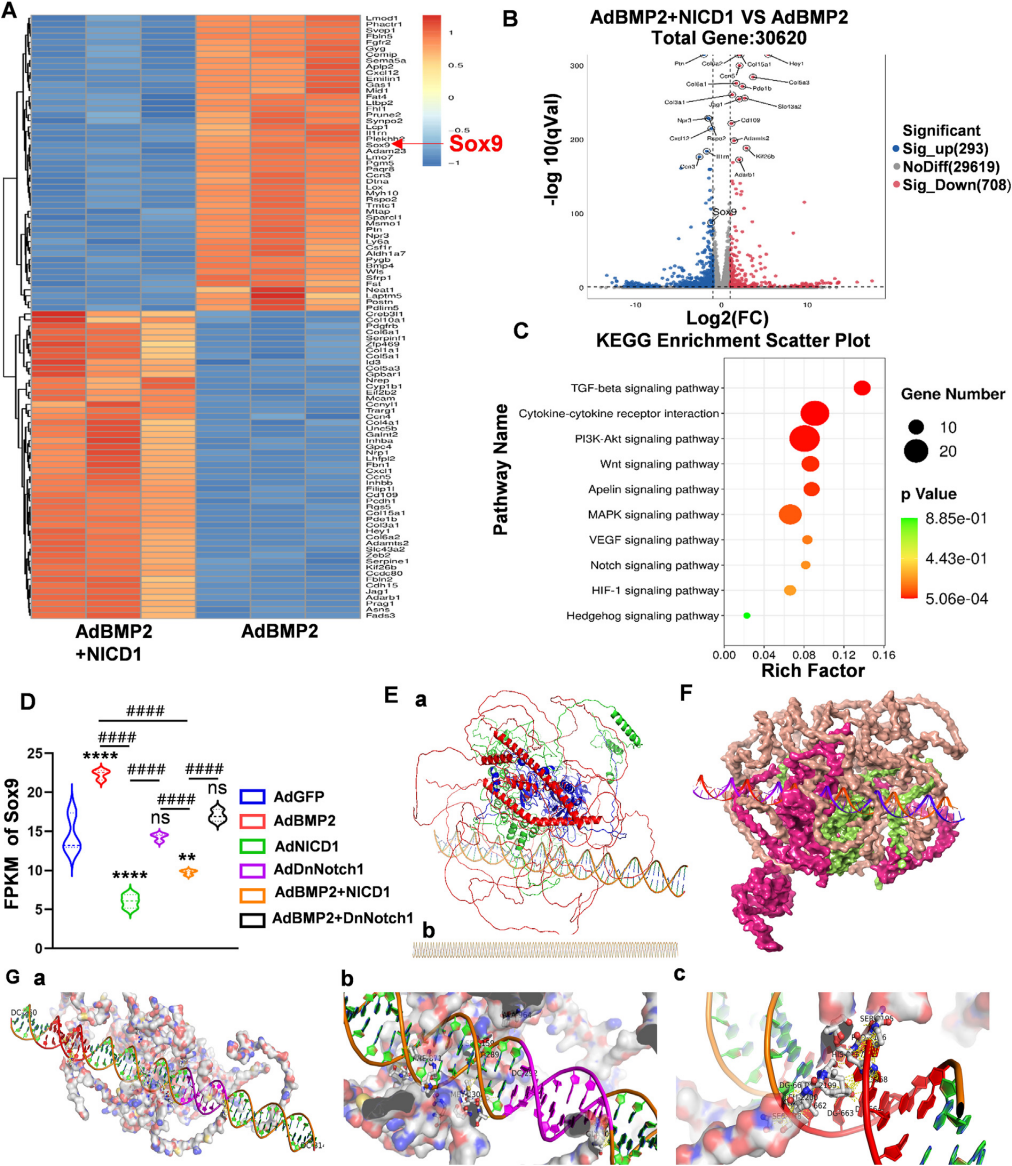
Sox9 and VEGFA were the potential targets for regulating the Notch1 signaling in BMP2-induced endochondral ossification. **(A)** Transcriptome sequencing was used for detecting potential targets. Between the AdBMP2 group and AdBMP2+AdNICD1 group, the top 100 differential genes were listed and the key chondrogenic differentiation transcription factor Sox9 was found. **(B)** Volcano maps of differential genes between the AdBMP2+AdNICD1 and AdBMP2 groups (293 genes up-regulated and 708 genes down-regulated). Differential genes with FC (fold change) ≥2 were accepted as positive, and the key chondrogenic differentiation transcription factor Sox9 was marked. **(C)** Pathway enrichment bubble map based on KEGG enrichment analysis. The enrichment factor indicated a higher degree of enrichment, a larger *p*-value (−log_10_) indicates a higher statistical significance, and a larger bubble indicates a higher degree of enrichment. Among them, the VEGFA signaling pathway was identified. **(D)** Fragments per kilobase of exon model per million mapped fragments (PFKM) analysis of Sox9 in each group indicated that activation of Notch1 signaling down-regulated Sox9 expression. **(E)** Molecular docking indicated the binding of RBPjk and Sox9 promoter. RBPjk-NICD1-MAML-1 protein complex **(*a*)** and Sox9 transcriptional promoter **(*b*)** (red for MAML-1 protein, green for NICD1 protein, blue for RBPjk protein) were shown. **(F)** Three-dimensional structure model and surface model of Sox9 promoter and RBPjk-NICD1-MAML-1 protein complex. **(G)** Segmental map of protein complex binding to Sox9 transcriptional promoter area (25 Å of amino acid residues close to DNA; Saci in red; Creb in purple) **(*a*)**. At the Saci site of DNA, SER2195 and PRO2196 of NICD1 bound with DC269 by forming hydrogen, HIS2197 with DC268, TYR2199 with DG663 and DG664, and LEU2200 with DA662 by forming hydrogen **(*b*)**. In the Creb region of DNA, SER 159 of RBPjk bound with DNA DT289 by forming hydrogen, and MET 2308 with DA642, VAL2309 with DC643 by forming hydrogen **(*c*)**. One-way analysis of variance; ∗∗∗∗*p* < 0.0001 and ∗∗*p* < 0.01 versus the AdGFP group; ^####^*p* < 0.0001 versus the indicated group; ns, *p* > 0.05. BMP2, bone morphogenetic protein 2; MAML-1, mastermind-like transcriptional coactivator 1; NICD1, Notch1 intracellular domain; Notch1, Notch receptor 1; RBPjk, recombination signal-binding protein for immunoglobulin kappa J region; Sox9; SRY-box transcription factor 9; VEGFA, vascular endothelial growth factor A.

**Figure 5:**
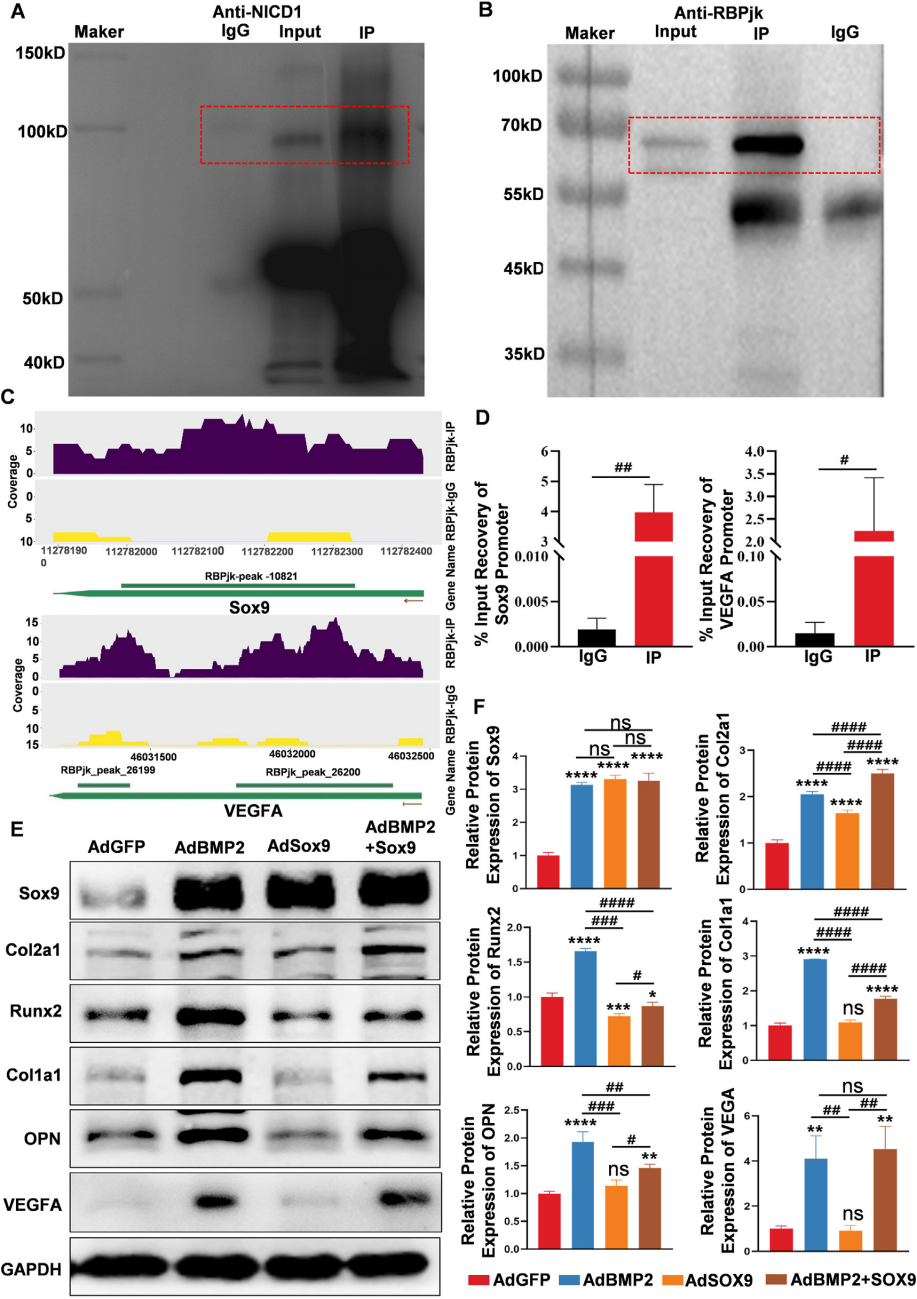
Notch1 signaling promoted BMP2-induced endochondral ossification by simultaneously down-regulating Sox9 transcription and increasing VEGFA expression. **(A)** The binding of NICD1 to RBPjk. The interaction between NICD1 and RBPjk was testified by co-immunoprecipitation. NICD1 protein was pulled down by RBPjk antibody, and the expression of NICD1 was detected by Western blot analysis after protein precipitation, taking IgG as the control group. **(B)** RBPjk mediated regulation of target gene expression. The RBPjk protein-DNA complex was pulled down by RBPjk antibody, the expression of RBPjk was identified in the Input group, taking IgG as the control group, RBPjk were detected by Western blot after protein precipitation (IP group), and RBPjk protein-DNA complex was subjected to CHIP-seq. **(C)** CHIP-seq indicated the binding of the RBPjk complex between Sox9 and VEGFA promoters. CHIP-Seq analysis showed the binding sites of Sox9 were in the promoter region from 112781993 to 112782331 **(*a*)** and the binding sites of VEGFA were in the promoter region from 46031275 to 46031455 and from 46031827 to 46032374 **(*b*)**. **(D)** Quantitative analysis of CHIP products. CHIP products were subjected to quantitative PCR analysis, the corresponding primers were designed according to the CHIP-Seq sites, and relative expression of the Sox9 promoter sequence and VEGFA promoter sequence were shown. **(E)** Sox9 promoted chondrogenic differentiation marker expression, inhibited endochondral ossification marker expression, and did not influence VEGFA expression. AdSox9 was used to overexpress Sox9 in BMP2-mediated chondrogenic differentiation. At indicated time points, Western blot assays were used for detecting the chondrogenic markers Sox9 and Col2a1, the endochondral ossification markers Runx2, Col1a1, and ONP, and the angiogenic differentiation marker VEGFA. Cropped blots were presented. **(F)** Quantitative analysis of Western blot results. Overexpression of Sox9 in BMP2-induced chondrogenic differentiation of MSCs promoted chondrogenic differentiation marker expression, inhibited endochondral ossification marker expression, and did not influence VEGFA expression. One-way analysis of variance; ∗∗∗∗*p* < 0.0001, ∗∗∗*p* < 0.001, ∗∗*p* < 0.01, and ∗*p* < 0.05 versus the AdGFP group; ^####^*p* < 0.0001, ^###^*p* < 0.001, and ^##^*p* < 0.01 versus the indicated group; ns, *p* > 0.05. BMP2, bone morphogenetic protein 2; CHIP-seq, chromatin immunoprecipitation followed by sequencing; Col1a1, collagen type I alpha 1 chain; Col2a1, collagen type II alpha 1 chain; MSC, mesenchymal stem cell; NICD1, Notch1 intracellular domain; Notch1, Notch receptor 1; RBPjk, recombination signal-binding protein for immunoglobulin kappa J region; Runx2, RUNX family transcription factor 2; Sox9; SRY-box transcription factor 9; VEGFA, vascular endothelial growth factor A.

As previously characterized, Sox9 is the key transcription factor for chondrogenic differentiation and inhibits Runx2 expression.^12^^,^^24^^,^^28^ Here, we confirmed that overexpression of Sox9 promoted the expression of the chondrogenic markers Sox9 and Col2a1, inhibited Runx2, Col1a1, and OPN expression, and did not influence VEGFA expression (Fig. 5E). Quantitative analysis showed the same trend (Fig. 5F).

**Fig. 6:**
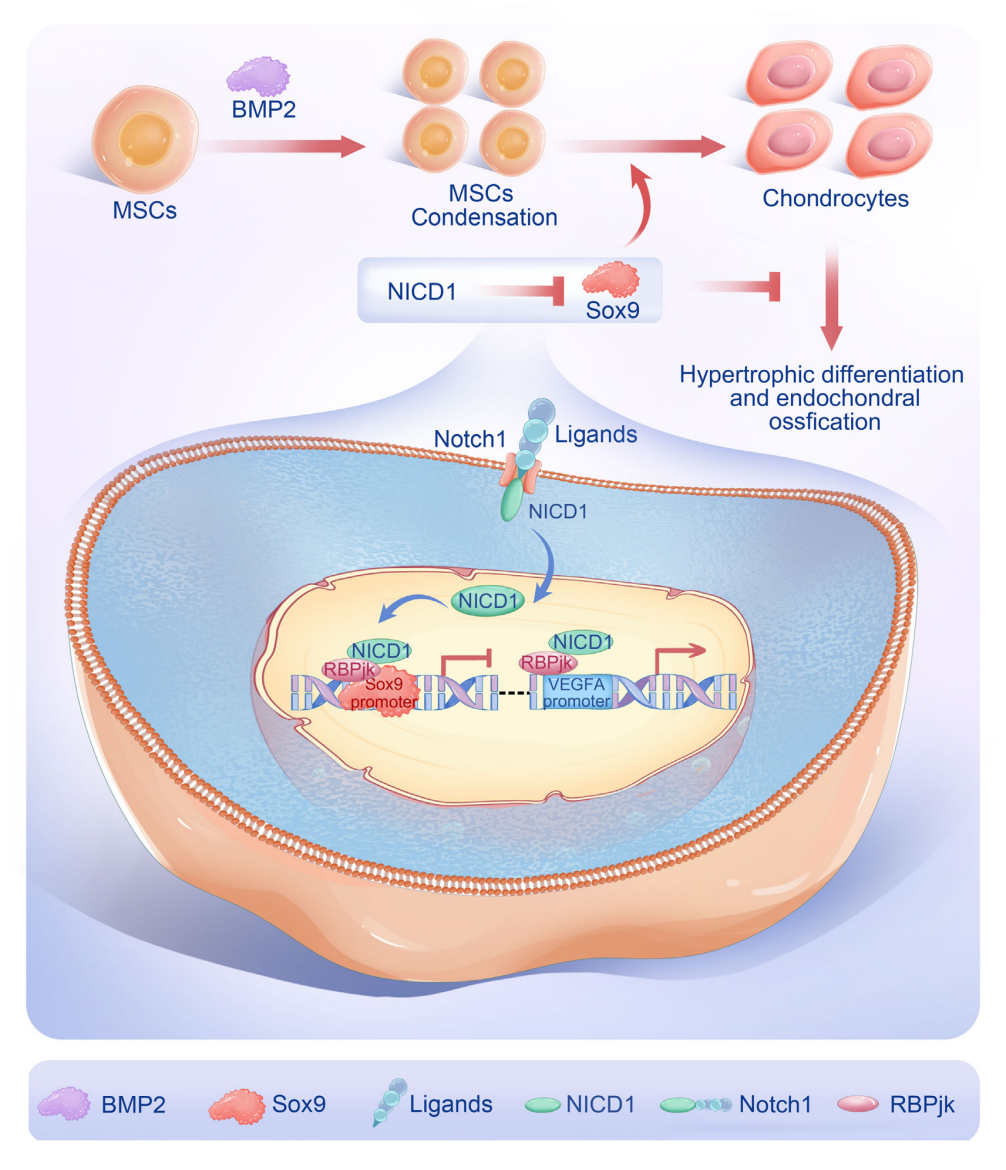
A mode diagram summarizing the main findings of the study. BMP2 induced MSC chondrogenic differentiation and triggered endochondral ossification. Sox9 promoted chondrogenic differentiation and inhibited BMP2-induced endochondral ossification. Notch1 regulated BMP2-induced endochondral ossification by inhibiting Sox9 expression and enhancing VEGFA expression. BMP2, bone morphogenetic protein 2; MSC, mesenchymal stem cell; Notch1, Notch receptor 1; Sox9; SRY-box transcription factor 9; VEGFA, vascular endothelial growth factor A.

Taken together, these findings suggest that activated Notch1 signaling can suppress Sox9 expression, leading to inhibition of osteogenic differentiation and promotion of chondrogenic differentiation. Additionally, Notch1 signaling can support angiogenic differentiation by up-regulating VEGFA expression (Fig. 6).

## Discussion

Cartilaginous pathologies present a great challenge for orthopedic surgeons due to their lack of regenerative capabilities.^42^ Currently, bone marrow stimulation and cartilage restoration are two primary clinical treatment methods.^43^^,^^44^ However, bone marrow stimulation such as microfracture and drilling, promotes fibrocartilage generation and is not suitable for large-size cartilage defects; restoration methods such as autologous chondrocyte implantation and osteochondral allograft are limited by insufficient cell supply and damage to donor site.^42^, ^43^, ^44^, ^45^, ^46^ Therefore, stem cell-based gene-enhanced cartilage regeneration is more promising for addressing these cartilaginous pathologies.^47^^,^^48^

BMP2 is one of the most potent growth factors which induce MSC chondrogenic differentiation. Mechanistically, Sox9 is the key transcription factor of BMP2-induced chondrogenic differentiation of MSCs.^13^ Additionally, we observed that Sox9 could inhibit BMP2-induced osteogenic differentiation of MSCs, thereby contributing to the chondrocytes maintaining their phenotype.^9^^,^^12^^,^^49^ However, the mechanisms of BMP2-triggered endochondral ossification of MSCs are far from clear. In the present study, to clarify the mechanisms of BMP2-induced endochondral ossification, we focus on the regulation function of Notch1 signaling on BMP2-induced differentiation of MSCs, the results showed that activation of Notch1 signaling promotes BMP2-induced osteogenic and angiogenic differentiation and inhibits BMP2-induced chondrogenic differentiation of MSCs. In mechanism, we found that Sox9 was one of the key differential genes with the activation of Notch1 signaling and activated Notch1 signaling inhibited the expression of Sox9 by RBPjk-dependent Sox9 promoter inactivation.

Articular cartilage covers the ends of long bone and is made up of hyaline cartilage, which lacks nerve, lymph vessel, and blood vessel distribution. Therefore, local cartilage injury is generally irreversible and deteriorates over time and results in osteoarthritis ultimately.^43^^,^^50^ Clinically, there is still no satisfactory therapeutic method for cartilage repair, highlighting the significant potential for stem cell-based approaches for this purpose.^43^^,^^44^^,^^46^^,^^48^ Although several cell source-derived MSCs have exhibited their great potential for cartilaginous tissue generation, terminal differentiation into hypertrophic chondrocytes, subsequently endochondral ossification, and finally replacement by osseous tissue.^47^^,^^48^ Therefore, elucidating the mechanisms of hypertrophic and endochondral ossification is essential for hyaline cartilage regeneration. As one of the most potent chondrogenic growth factors, BMP2 holds the potential to induce MSC chondrogenic differentiation, however, BMP2-induced hypertrophic and endochondral differentiation of MSCs still the biggest drawback of BMP2-mediated cartilage regeneration. We have previously characterized that Runx2 is the key transcription factor regulating BMP2-induced osteogenic and hypertrophic differentiation, and that overexpression of Sox9 could promote BMP2-induced chondrogenic and inhibit BMP2-induced osteogenic differentiation of MSCs.^30^ Here, we identified that Notch1 signaling regulated BMP2-induced osteogenic, chondrogenic, and angiogenic differentiation of MSCs. Furthermore, Notch1-mediated regulation of Sox9 determines the fate of MSC chondrogenic or osteogenic differentiation, which is in accordance with the function of Notch signaling in bone development.^18^

Notch signaling is a highly conserved pathway that controls cell fate decisions. Activated Notch signaling is based on the ligand-activated receptors between adjacent cells.^51^ During bone development, the activation of Notch signaling is indispensable for angiogenesis and osteogenesis-angiogenesis coupling.^52^ As for chondrogenesis and cartilage development, Notch signaling is also involved in cell lineage determination processes by regulating Sox9 expression. Temporary activation of Notch1 during the early stages of the embryoid body results in induction of chondrogenic differentiation, however, continuous activation of Notch1 activation could result in complete inhibition of chondrogenic differentiation.^7^ In addition, Dong Y et al have determined that the RBPjk-dependent Notch signaling pathway is a crucial regulator of MSC proliferation and differentiation during skeletal development.^53^ In mechanism, Chen S et al found that with the activation of Notch1 signaling, the RBPjk/NICD transcription complex binding site is the upstream of Sox9 promoter, in other words, Notch negatively regulates chondrocyte differentiation in the axial skeleton by suppressing Sox9 transcription.^8^ Here, our data suggested that Notch signaling was activated in the early stage of BMP2-induced chondrogenic differentiation of MSCs, and exogenous activation of Notch1 inhibited BMP2-induced chondrogenic and potentiated osteogenic differentiation of MSCs. Simultaneously, activated Notch1 signaling promoted angiogenesis of endochondral ossification by promoting VEGFA expression. These results indicate that Notch1 signaling determines cell lineage of BMP2-induced MSCs osteogenic or chondrogenic differentiation and regulates endochondral ossification.

Sox9 is the key transcript factor involved in the chondrogenic differentiation of MSCs. It is well-characterized that Sox9 not only governs chondrogenic differentiation of MSCs but also keeps growth plates and articular cartilage healthy by inhibiting chondrocyte dedifferentiation or osteoblastic redifferentiation.^54^ Several studies have investigated Notch1-Sox9 regulation during chondrogenic differentiation and endochondral ossification of bone development; it is not clear whether Notch1 regulates Sox9 during BMP2-induced chondrogenic differentiation of MSCs.^8^^,^^53^^,^^55^ Here, we identified that the RBPjk-NICD1 transcription complex could inhibit Sox9 expression in BMP2-induced chondrogenic differentiation of MSCs. Besides, Notch1-Sox9 regulation may be the core regulation for lineage determination and chondrocyte phenotype maintenance in BMP2-mediated bone and cartilage tissue engineering.

Although cartilage regeneration poses a significant challenge, it holds immense potential for effectively treating cartilage injuries. In the present study, we found the mechanisms of BMP2-induced endochondral ossification were regulated by Notch1 signaling. Activated Notch1 signaling could inhibit the expression, thereby hindering the maintenance of the chondrocyte phenotype. Conversely, activated Notch1 signaling could promote osteogenic and angiogenic differentiation, thereby facilitating endochondral ossification. Collectively, Notch1 signaling has emerged as a pivotal signaling pathway mediating the maintenance of BMP2-induced cartilage regeneration in MSCs.

In conclusion, activated Notch1 signaling can promote BMP2-induced endochondral ossification of MSCs by down-regulating Sox9-mediated chondrogenic differentiation and promoting VEGFA-mediated angiogenesis.

## CRediT authorship contribution statement

Conception and design: Wei Huang and Junyi Liao; analysis and interpretation of the data: Jing Zou, Chengcheng Du, Senrui Liu, Shengqiang Gao, and Bowen Chen; drafting of the manuscript: Junyi Liao and Jing Zou; critical revision of the manuscript for important intellectual content: Junyi Liao and Wei Huang; provision of study materials: Junyi Liao, Zhenglin Zhu, and Wei Huang; statistical expertise: Jing Zou; obtaining of funding: Junyi Liao, Zhenglin Zhu, and Wei Huang; administrative, technical, or logistic support: Junyi Liao and Wei Huang; collection and assembly of data: Jing Zou, Junyi Liao, Senrui Liu, and Chengcheng Du; final approval of the manuscript: all authors.

## Funding

The reported work was supported by the Science and Technology Research Program of the Chongqing Education Commission (China) (No. KJQN202100431, KJZD-M202100401). This project was also supported by the National Natural Science Foundation of China (No. 81972069, 82002312), CQMU Program for Youth Innovation in Future Medicine (Chongqing, China) (No. W0154), Innovation Project from Chongqing Municipal Education Commission (China) (No. CYB21169), Cultivating Program and Candidate of Tip-Top Talent of The First Affiliated Hospital of Chongqing Medical University (Chongqing, China) (No. BJRC2021-04), Cultivating Program of Postdoctoral Research of The First Affiliated Hospital of Chongqing Medical University (Chongqing, China) (No. CYYY-BSHPYXM-202202), Special Support from Chongqing Postdoctoral Research Program (Chongqing, China) (No. 2021XM1029). J.Y. was supported by a postdoctoral fellowship from Chongqing Medical University and rewarded by China Postdoctoral Science Foundation (No. 2022M720605). Funding sources were not involved in the study design, in the collection, analysis, and interpretation of data; in the writing of the report; and in the decision to submit the paper for publication.

## Ethics declaration

All research protocols in this study (Notch1 signaling regulates BMP2-induced endochondral ossification of MSCs through angiogenesis signaling) were approved by the Ethics Committee of the First Affiliated Hospital of Chongqing Medical University (March 3rd, 2022, 2022-K92).

## Data availability

All datasets generated for this study are included in the article and supplementary materials.

## Conflict of interests

The authors declared no conflict of interests.

## Figures and Tables

**figs1:**
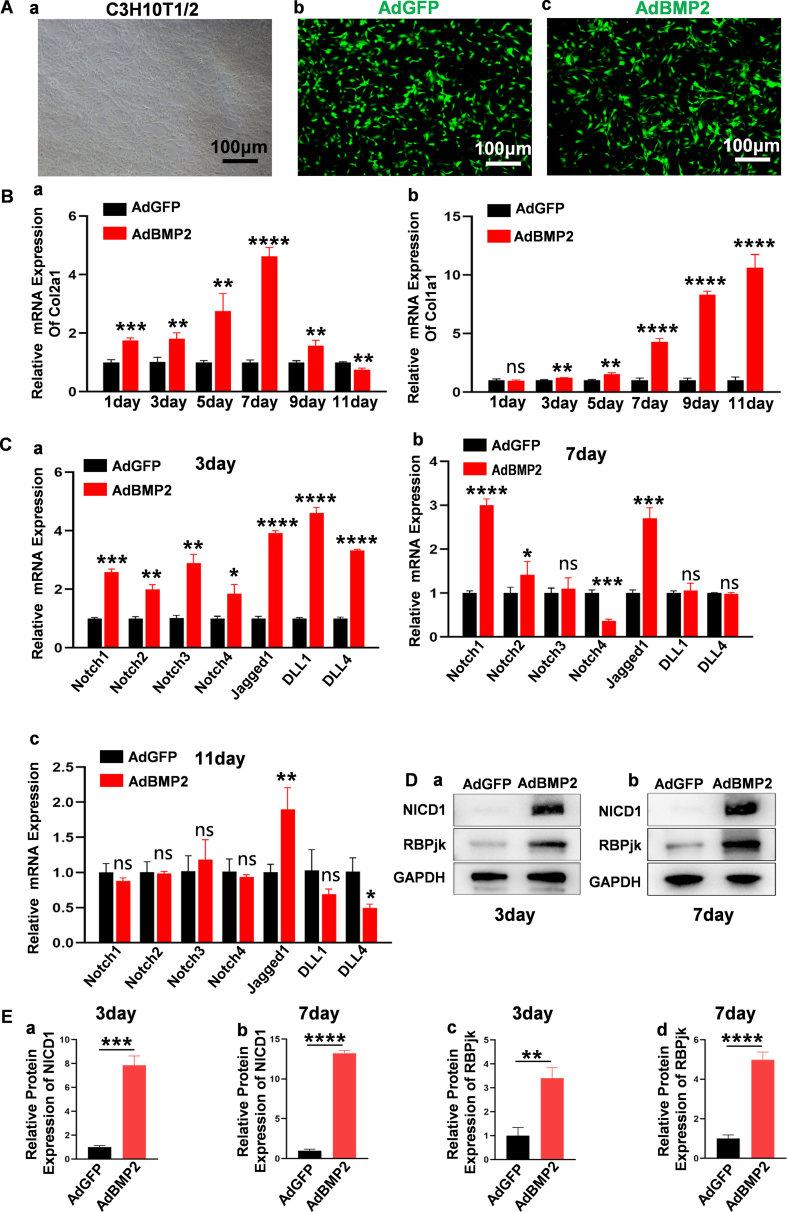


**figs2:**
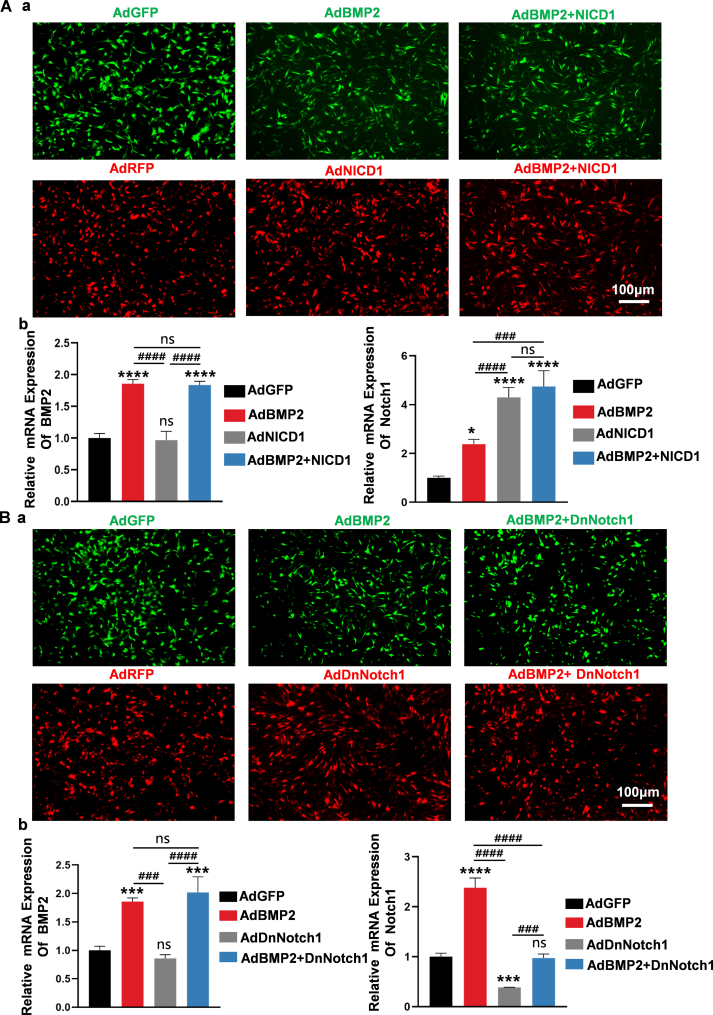


**figs3:**
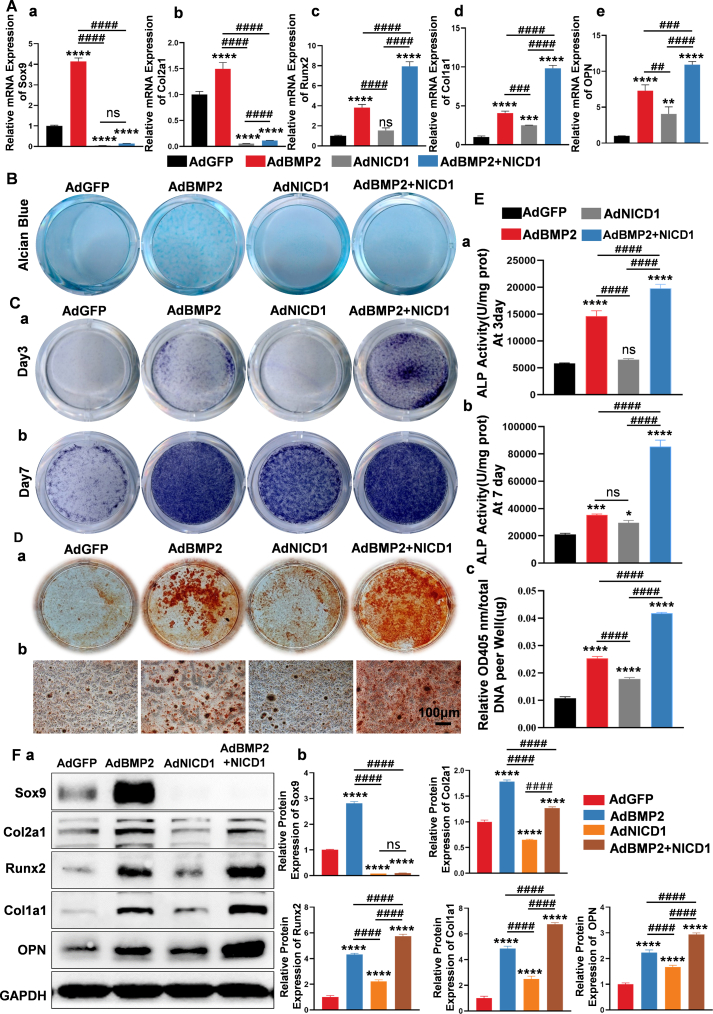


**figs4:**
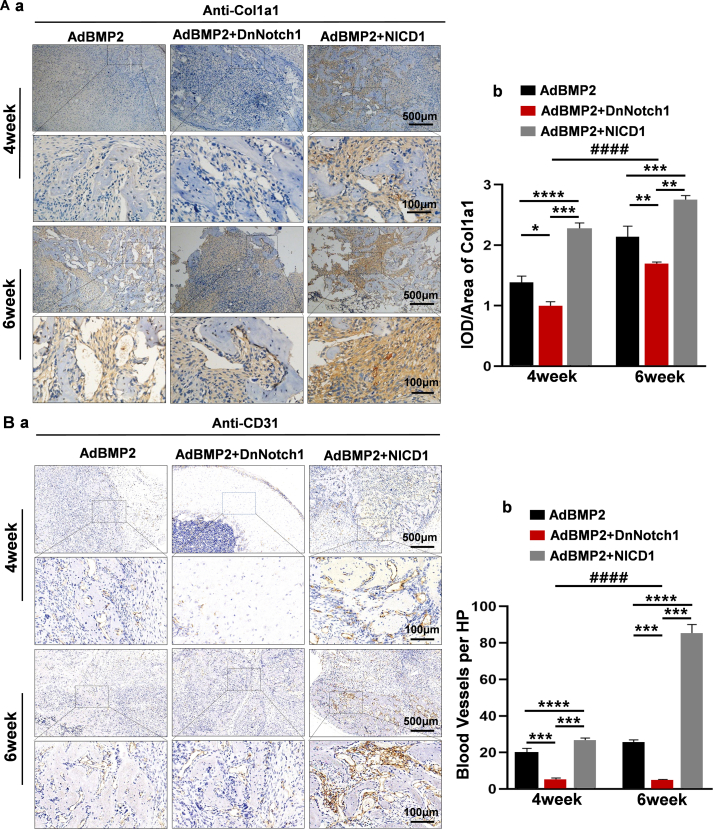


**figs5:**