# Supplementary material for: Notch1 signaling regulates Sox9 and VEGFA expression and governs BMP2-induced endochondral ossification of mesenchymal stem cells
Source: Genes Dis. 2024 May 20;12(3):101336. doi: 10.1016/j.gendis.2024.101336 (PMC11905894; doi:10.1016/j.gendis.2024.101336)
Supplement: Multimedia component 1 [file mmc1.docx]

**Figure S1** The activation of Notch signaling in BMP2-induced MSC differentiation. **(A)** C3H10T1/2 cells were transfected with AdBMP2 and AdGFP respectively. Two days later, the bright field **(*a*)** and green fluorescent protein (GFP) fluorescence field **(*b*, *c*)** were recorded. Scale bar =100 μm. **(B)** BMP2-induced chondrogenic and osteogenic marker expression of MSCs. The relative expression levels of Col2a1 mRNA **(*a*)** and Col1a1 mRNA **(*b*)** were detected by quantitative reverse transcription PCR on day 1, day 3, day 5, day 7, day 9, and day 11, taking AdGFP as a control group. **(C)** BMP2-induced expression of Notch receptors and ligands of MSC differentiation. The relative expression levels of Notch receptors (Notch1–4) and its ligands (Jagged1, DLL1, and DLL4) were detected by quantitative reverse transcription PCR on day 3 **(*a*)**, day 7 **(*b*)**, and day 11 **(*c*)** after BMP2 stimulation. **(D, E)** BMP2-induced activation of Notch1 signaling. (D) NICD1 and RBPjk were detected by western blot and BMP2 dramatically promoted NICD1 expression on day 3 **(*a*)** and day 7 **(*b*)**. (E) Quantitative analysis of relative expression of NICD1 **(*a*)** and RBPjk **(*b*)**. One-way analysis of variance and *t*-tests; ^****^*P* < 0.0001, ^***^*P* < 0.001, ^**^*P* < 0.01, and ^*^*P* < 0.05 versus the hAdGFP group; ns, *P* > 0.05. Notch1, Notch receptor 1; BMP2, bone morphogenetic protein 2; MSC, mesenchymal stem cell; NICD1, Notch1 intracellular domain; Col2a1, collagen type II alpha 1 chain; Col1a1, collagen type I alpha 1 chain; RBPjk, recombination signal-binding protein for immunoglobulin kappa J region; DLL1/4, Delta 1/4.

**Figure S2** Adenovirus-mediated target gene expression. **(A)** AdNICD1 effectively up-regulated Notch1 signaling in BMP2-induced MSC differentiation. C3H10T1/2 cells were infected with AdBMP2, AdNICD1, AdBMP2+NICD1, AdGFP, and AdRFP were used as control. Three days later, the fluorescence field of GFP (AdGFP, AdBMP2, AdBMP2+NICD1) and RFP (AdRFP, AdNICD1, AdBMP2+NICD1) were recorded **(*a*)**. The fluorescence showed high efficiency in single or combined infection rate of adenovirus, scale bar = 100 μm. Quantitative PCR was applied for detecting BMP2 and NICD1 mRNA expressions **(*b*)**. **(B)** AdDnNotch1 effectively down-regulated Notch1 signaling activation in BMP2-induced MSC differentiation. C3H10T1/2 cells were infected with AdBMP2, AdDnNotch1, and AdBMP2+DnNotch1; AdGFP and AdRFP were used as controls. Three days later, the fluorescence field of GFP (AdGFP, AdBMP2, AdBMP2+ DnNotch1) and RFP (AdRFP, Ad DnNotch1, AdBMP2+DnNotch1) were recorded **(*a*)**. The fluorescence showed high efficiency in single or combined infection rates of adenovirus. Scale bar = 100 μm. Quantitative PCR was applied for detecting BMP2 and NICD1 mRNA expression **(*b*)**. One-way analysis of variance; ^****^*P* < 0.0001, ^***^*P* < 0.001, and ^*^*P* < 0.05 versus the AdGFP group; ^####^*P* < 0.0001 and ^###^*P* < 0.001 versus the indicator group; ns, *P* > 0.05. Notch1, Notch receptor 1; BMP2, bone morphogenetic protein 2; MSC, mesenchymal stem cell; NICD1, Notch1 intracellular domain.

**Figure S3** Up-regulation of Notch1 signaling inhibited BMP2-induced chondrogenic differentiation and promoted BMP2-induced osteogenic differentiation of MSCs *in vitro*. **(A)** C3H10T1/2 cells were transfected with AdGFP, AdBMP2, AdNICD1, and AdBMP2+NICD1, separately. On day 7, quantitative reverse transcription PCR was used to detect the expression of chondrogenic differentiation markers (Sox9 and Col2a1) and osteoblastic differentiation markers (Runx2, Col1a1, and OPN) of MSCs. **(B)** To detect the expression of sulfated glycosaminoglycan during C3H10T1/2 cell differentiation, alcian blue staining was performed on day 7 after cells were transfected with recombinant adenovirus. **(C)** Alkaline phosphatase (ALP) staining experiments were used to determine ALP activity on day 3 **(*a*)** and day 7 **(*b*)**. **(D)** For matrix mineralization, alizarin red S staining was performed on day 14 **(*a*)**; microscopic **(*b*)** observations showed that up-regulation of Notch1 signaling promoted BMP2-induced calcium deposition. **(E)** Quantitative analysis of ALP activities and calcium deposition. The ALP activity was quantified at OD 405 nm and normalized by protein concentration per well (unit/mg protein) on day 3 **(*c*)** and day 7 **(*d*)**. Alizarin Red staining was quantified at OD 405 nm and normalized to total DNA per well (OD 405 nm/ug DNA) **(*c*)**. **(F)** Western blot analysis for chondrogenic differentiation markers (Sox9 and Col2a1) and osteogenic markers (Runx2, Col1a1, and OPN). Protein bands **(*a*)** and quantitative analysis **(*b*)**. The relative expression of Sox9, Col2a1, Runx2, Col1a1, and OPN proteins were analyzed using GAPDH as a control **(*b*)**. One-way analysis of variance; ^****^*P* < 0.0001, ^***^*P* < 0.001, ^**^*P* < 0.01, ^*^*P* < 0.05 versus the AdGFP group; ^####^*P* < 0.0001, ^###^*P* < 0.001, ^##^*P* < 0.01, and ^#^*P* < 0.05 versus the indicated group; ns, *P* > 0.05. Notch1, Notch receptor 1; BMP2, bone morphogenetic protein 2; MSC, mesenchymal stem cell; NICD1, Notch1 intracellular domain; Sox9, SRY-box transcription factor 9; Col2a1, collagen type II alpha 1 chain; Runx2, RUNX family transcription factor 2; Col1a1, collagen type I alpha 1 chain; OPN, osteopontin.

**Figure S4** Notch1 signaling regulated BMP2-induced endochondral ossification *in vivo*. **(A)** Col1a1 stands for osteogenic activity, up-regulation of Notch1 signaling with AdNICD1 increased Col1a1 positive area, and down-regulation of Notch1 signaling with AdDnNotch1 decreased Col1a1 positive area compared with the AdBMP2 group at 4 weeks and 6 weeks. The scale bar is 100 μm in a low power field or 500 μm in a high-power field **(*a*)**. Quantitative analysis of positive Col1a1 showed that NICD1 enhanced the BMP2-induced osteogenic differentiation ability of MSCs and AdDnNotch1 inhibited the BMP2-induced osteogenic differentiation ability of MSCs compared with the AdBMP2 group **(*b*)**. **(B)** CD31^+^ cells stand for the angiogenesis process, up-regulation of Notch1 signaling with AdNICD1 increased new blood vessel formation, and down-regulation of Notch1 signaling with AdDnNotch1 decreased new blood vessel formation compared with the AdBMP2 group at 4 weeks and 6 weeks. The scale bar is 100 μm in a low power field or 500 μm in a high-power field **(*a*)**. Quantitative analysis of new blood vessels showed that NICD1 enhanced the BMP2-induced new blood vessel formation of MSCs and AdDnNotch1 inhibited BMP2-induced new blood vessel formation of MSCs compared with the AdBMP2 group **(*b*)**. One-way analysis of variance; ^****^*P* < 0.0001, ^***^*P* < 0.001, ^**^*P* < 0.01, and ^*^*P* < 0.05 versus the AdBMP2 group. Two-way analysis of variance; ^####^*P* < 0.0001 versus the 4-week group. Notch1, Notch receptor 1; BMP2, bone morphogenetic protein 2; MSC, mesenchymal stem cell; NICD1, Notch1 intracellular domain; Col1a1, collagen type I alpha 1 chain.

**Figure S5** Notch1 signaling regulated BMP2-induced HUVEC proliferation and migration. **(A)** HUVECs and MSCs induced by indicated adenovirus were co-cultured in the Transwell system. The HUVEC proliferation capacity was measured by EDU proliferation assay, and the stained cells were recorded 24 h later. Scale bar = 200 μm. **(B)** HUVECs and MSCs induced by indicated adenovirus were co-cultured in the Transwell system. The migratory ability of HUVECs was evaluated by wound-healing experiments (recorded 0 and 12 h after scratching). Scale bar = 200 μm. **(C)** Quantitative analysis of the number of proliferating cells **(*a*)** and migration distance **(*b*)**. One-way analysis of variance; ^****^*P* < 0.0001, ^***^*P* < 0.001, and ^**^*P* < 0.01 versus the AdGFP group; ^####^*P* < 0.0001 and ^###^*P* < 0.001 versus the indicator group; ns, *P* > 0.05. Notch1, Notch receptor 1; BMP2, bone morphogenetic protein 2; MSC, mesenchymal stem cell; HUVEC, human umbilical vein endothelial cell.

**Table S1** Primer oligonucleotide sequences used for PCR.

| **Genes (mouse)** | **Forward** | **Reverse** |
| --- | --- | --- |
| GAPDH | CCTCGTCCCGTAGACAAAATG | TGAGGTCAATGAAGGGGTCGT |
| Sox9 | GGACATCGGTGAACTGAGCA | GCGTGACTGTAGTAGGAGCC |
| Col2a1 | GCCAGGATGCCCGAAAATTAG | CGTCATACCCTCCAGCCATC |
| Runx2 | AGCGGACGAGGCAAGAGTTT | AGGCGGGACACCTACTCTCATA |
| Col1a1 | GAGAGGTGAACAAGGTCCCG | AAACCTCTCTCGCCTCTTGC |
| OPN | TGGCTGAATTCTGAGGGACTAA | GCAGGCTGTAAAGCTTCTTCTCC |
| Notch1 | TGGACTGTTCTGTGCATCCC | TGGGGATCAGAGGCCACATA |
| Notch2 | AGCAGGAGGGGCAGGTAG | GGTTCGCTCAGCAGCATT |
| Notch3 | CTGGCTCCAGATGCCTGT | GGGGACAGCACCTCACAC |
| Notch4 | CCGTCCTGGTTTCACAGG | GACTTCCGTCAGGGCAGA |
| DLL1 | CCGGTTTGTGTGTGACGA | CCAGGGTCGCACATCTTC |
| DLL4 | GGGCCTTCCTTCTGCATT | ACTCTTGGCGGGTTCACA |
| Jag1 | CCAACACGGTCCCCATTA | TTGGCAAAGCGGACTTTC |
| Sox9-promoter | CGTGATTGGCCCGAGGTATC | AAAGTTGTCGCTCCCACAGA |
| VEGFa-promoter | TCCCCTCTTAAATCGTGCCA | CTGCTGATTTCCACAATCCGA |
